# The Forgotten Age Phase of Healthy Lifestyle Promotion? A Preliminary Study to Examine the Potential Call for Targeted Physical Activity and Nutrition Education for Older Adolescents

**DOI:** 10.3390/ijerph19105970

**Published:** 2022-05-14

**Authors:** Kristy Howells, Tara Coppinger

**Affiliations:** 1 Department of Sport, Exercise and Rehabilitation Sciences, School of Psychology and Life Sciences, Faculty of Science, Engineering and Social Sciences, Canterbury Christ Church University, Canterbury CT1 1QU, UK; 2 Department of Sport, Leisure and Childhood Studies, Faculty of Business and Humanities, Munster Technological University, T12 P928 Cork, Ireland; tara.coppinger@mtu.ie

**Keywords:** physical activity, nutrition, healthy lifestyles, health promotion, health behaviours, adolescent voice, older adolescents, high school, health literacy

## Abstract

To date, little research on healthy lifestyle promotion has focused on older adolescents (16–18-year-olds), yet this is a key time that habitual healthy lifestyles could be developed. Ninety-three participants (thirty-nine males; fifty-four females) (mean age = 16.9, (SD 0.4) years), from three low socio-economic high schools in England, completed an online questionnaire on their self-reported: (i) daily physical activity (PA), (ii) active transportation, (iii) active leisure time, (iv) food intake and (v) experiences of how healthy lifestyles are promoted specifically to them. Overall, 60% reached the daily PA recommended guidelines. Yet, 92% used a bicycle/walked for a least 10 min continuously as active transport and of these, 86% undertook this at least 5 days per week. Almost half undertook MVPA as active leisure, but 66% still spent ≥ 5 h sedentary. Seventeen percent met recommended nutritional guidelines for health and 90% (*n* = 80) did not report school as a place that promoted healthy lifestyles. It is recommended as a public health measure and as an educational policy matter that schools implement more targeted PA and healthy eating initiatives for older adolescents that also include the adolescent voice. Further, gaining a deeper insight into male older adolescents’ health literacy is needed.

## 1. Introduction

To date, little research on healthy lifestyle promotion has focused on older adolescents (16–18-year-olds), particularly in the UK, yet this is a key time that habitual healthy lifestyles could be developed. The purpose of this preliminary study was to examine older adolescents’ knowledge and understanding of England’s healthy lifestyle recommendations; specifically, the recommended physical activity (PA) and nutrition guidelines, as well as their experiences of how healthy lifestyles are promoted to them.

Adolescence has been identified as a key time when habitual health behaviours can be learnt that track into adulthood; specifically related to exercise and food [1]. Yet, globally, there has been a gap in healthy lifestyle promotion and associated scientific research among older adolescents, with most campaigns having previously been tailored for younger children or adults [2]. Further, the World Health Organization Europe [3] found that only eight countries involved the child and adolescent voice in the review, development, and implementation of health strategies. Ultimately, raising concerns about the meaningfulness of health strategies aimed at this population.

From an educational perspective in England, 11–16-year-olds are educated about healthy lifestyles through the new Relationships Education, Relationships and Sex Education and Health Education curriculum that became the compulsory statutory curriculum in England in 2019 [4]. As a result, by the time students complete secondary/high school (secondary and high school runs from ages of 11–16 years), they are expected to understand healthy eating, particularly, “how to maintain healthy eating and the links between a poor diet and health risks” [4] (p. 37). They should also have a knowledge of physical health and fitness and, “the characteristics and evidence of what constitutes a healthy lifestyle” [4] (p. 37), as well as, “the positive associations between PA and the promotion of mental wellbeing” [4] (p. 37).

International researchers [5] have previously highlighted the importance of health education within the classroom and have stated that the development of adolescent health literacy, where students can “access, understand, evaluate and communicate health information” [5] (p. 350) is best developed during school hours. Schools have also been recommended as key places for food education, as childhood is a crucial period for developing eating habits that continue into adulthood [6]. Yet, the authors [6] went on to state that the current English curriculum does not advance food knowledge and skills across the primary age phases; instead, it gives a “scant, broad” approach to food education in the curriculum [6] (p. 18). This may be due to food and health education being split between areas of the curriculum. The Design and Technology curriculum includes cooking and nutrition [7], whilst the Relationships Education, Relationships and Sex Education and Health Education curriculum focuses on healthy eating and the links between planning and preparing healthy meals, as well as the risk of unhealthy eating [4]. With this disconnect, it is therefore not surprising that research has indicated England is approximately a decade behind other countries, such as Canada, in terms of recognising the importance of health education [8].

Health literacy can be regarded as a measurable outcome of health education [9] and research suggests that initiatives to reduce disparities in health education may benefit from a focus on health literacy [10]. The National Health Service [11] refers to the World Health Organization’s definition of health literacy as, “the personal characteristics and social resources needed for individuals and communities to access, understand, appraise and use information and services to make decisions about health” [11]. Yet, this definition has been considered too generic to whole populations, with no current, official definition of health literacy in existence for children or adolescents [12].

Having health literacy skills is important for maintaining or improving health [10], with low health literacy linked to a range of poor health outcomes [13]. Barriers to accessing health information, which would include content relating to PA and healthy eating [14], have been highlighted as important contributors. Since it has been assumed that the health (information) needs of adolescents are the same as those of adults [15], these young people may be missing out on opportunities to develop their health literacy, particularly as health education has only recently been introduced to the English curriculum [4].

In the developed world, there is now concern that life expectancy is dropping. This, in part, is directly linked to the rise of chronic diseases such as diabetes and early-onset cardiovascular disease, which are emerging in adolescence and early adulthood [16]. Poor intake of fruit and vegetables has been recognised as an important modifiable risk factor contributing to these conditions [17], with a strong protective mechanism shown between fruit and vegetable consumption and a reduced risk of hypertension, coronary heart disease, and stroke [18]. Yet, the National Diet and Nutrition Survey (NDNS) found only 8% of young people in the UK aged 11–18 years meet the recommended consumption of five or more portions of fruit and vegetables per day [19].

Globally, only one in five adolescents meet the PA guidelines of 60 min of moderate to vigorous physical activity (MVPA) per day and one in six adolescents are already overweight [20]. Coinciding with poor fruit and vegetable intakes, these young people are putting themselves at an even greater risk of mortality from lifestyle-related diseases [18]. The World Health Organization Europe [20] proposes that in order to promote habitual daily PA among adolescents, increasing opportunities for school-based PA, active transportation, and active leisure should be made a priority in countries.

Although a lack of PA, overeating, and poor dietary choices tend to cluster [21,22], older adolescents have generally been given little health or nutrition attention [23]. Their health needs are traditionally not considered by many countries [16], even though there may be adolescent-specific priority issues that call for specific strategies and approaches. One example from schools in England found most leaders in the schools did not feel accountable for health promotion [24]. There was also a limited understanding of which initiatives might be most effective within schools, as well as difficulty finding information about health promotion [24]. Consequently, these young people may not know, or fully understand, the importance of healthy lifestyle promotion or the direct impact that poor lifestyle behaviours may have on their lives.

Based on the review of the literature outlined within the introduction, it is hypothesised that older adolescents (16–18-year-olds) will have limited knowledge and understanding of healthy lifestyle (PA and nutrition) recommendations for their age group. This age bracket of older adolescents within the study would be amongst the first secondary school students within England to experience the new English Health Education curriculum. By including these participants, this study allows for a unique examination of how this new curriculum has impacted the knowledge and understanding of healthy lifestyles among older adolescents. This study aims to examine English older adolescents’ current knowledge and understanding of healthy lifestyles; specifically, their self-reported daily PA, engagement in active transportation and active leisure, as well as their food intake. How they feel healthy lifestyles are promoted to them will also be investigated. Comparisons will then be made between their knowledge and understanding of PA and nutrition recommendations and their current lifestyle behaviours.

## 2. Materials and Methods

### 2.1. Context

This research was set up as a bespoke research project to be offered as part of the Nuffield Foundation Research Placement Programme 2021. Due to the bespoke nature of the research, and the targeted socio-economic and geographical focus of the sample, this research aims to act as a preliminary study to examine if there is a need for more targeted PA and nutrition information for older adolescents, with the aim of upscaling to a larger sample of schools and adolescents in the future. The Nuffield Foundation Research Placement Programme is designed to provide engaging, hands-on research experience for talented year 12 students (those aged 17–18 years old) via engagement in meaningful, real-life research projects in science, quantitative social science, computing, technology, engineering, mathematics or a combination of areas. The research placement is over a 6-week period during the summer holidays. The students act as research assistants but due to COVID-19 restrictions, this work was undertaken virtually through Google classrooms and independent home working in 2021.

The students had to apply to be a part of the programme and had to meet set eligibility criteria set by Nuffield Foundation. These criteria included: (i) being in full-time state-funded education in the UK; (ii) having at least 5 GCSEs at level 6 or Scottish Nationals at level B in maths, science, English and another humanities subject; and (iii) currently studying in one or more A Levels or Scottish Highers in science, technology, engineering, economics, geography, geology, psychology, computer, statistics or mathematics. In terms of family socioeconomic status, the eligibility focused on prioritising students from low-income backgrounds. Students who met one or more of the following additional criteria were therefore prioritised: (i) students of families who had a combined income of less than £30,000; (ii) those entitled to free school meals or had received them within the last 6 years; (iii) students currently living or had previously lived in local authority care; (iv) if a student would be the first in their family to be applying to go to into higher education; (v) if the students were in entitled to bursary or school funding.

Three research assistants met the specified criteria and were matched to the research project via their personal statements in their application by the South East England Regional Coordinator. As part of the Nuffield Research Placement, the students had to complete a pre-placement virtual research module that introduced them to research methodology. As part of the experience of the placement, they also had to complete their own research poster and research report write-up that was tailored to one topic of their choice. These write-ups were separate from the findings reported in this current paper; however, the data collected and analysed is reported and discussed.

All three of the research assistants were virtually introduced to the lead researcher of the project via Google classrooms. During this meeting, they were guided through the purpose of the project, the current gap in knowledge within the field, and the questionnaires that were to be used within the project. In order to make the project meaningful, each of the 3 schools that the research assistants attended was purposefully targeted via the distribution of an online questionnaire. This enabled the research assistants to investigate their own setting, as well as others that were of a similar geographic and socioeconomic status (SES).

### 2.2. Participants and Their Schools

A total of 93 older adolescents (39 males and 54 females (mean age = 16.9, (SD 0.4) years) from three low socio-economic high schools (as derived from the National Census) [25] in South East England participated in this study. A purposeful sampling technique [26] was applied to the school sample to ensure older adolescents from the same SES and local region could be compared. Two participants did not complete the whole of the questionnaire and therefore their data was removed.

All older adolescents participating were studying for either Advanced Levels (A Levels) or the International Baccalaureate (IB) examinations, which are common pathways for students aged 16–18 years attending schools in England. The target sample for the study included the average class size of older adolescents in English schools (*n* = 22) [27]; which amounted to a minimum of 66 (1 class for each of the 3 schools) older adolescents (aged 16–18 years) in total. As there is some variation in ages within, and across, a year group, more students were able to be available to participate in the study, as they were within the study’s required age range.

Each of the schools had achieved a “Healthy School Status Mark’”, which aims to seek to achieve healthy lifestyles for the entire school population [28]. The schools follow the “Healthy Schools” programme [28] and the food served in their canteens complies with the Government’s Healthy Eating Guidelines [29]. The schools emphasise on their web pages that they encourage students to bring only healthy foods into school. They specify no fizzy drinks are allowed at any time, and all savoury snacks and chocolate bars are banned except for on Fridays, when they can bring a “treat”. In the Government-run inspection reports (Ofsted) for each of the schools, all of them achieved a good or broadly average rating for the extent to which learners adopt healthy lifestyles. Additional findings from these reports state that students understand the need for healthy lifestyles but not many were choosing fruit or salads within the options in the canteen.

### 2.3. Online Questionnaire and Ethical Considerations

The questions within the questionnaire were based upon previously validated questionnaires that have been used to examine PA and food intake across the age phases. The questionnaires were the Global Physical Activity Questionnaire (GPAQ) [30] and the Short Form Food Frequency Questionnaire (FFQ) [31]. The GPAQ was validated [32] as providing reproducible data and deemed a suitable and acceptable instrument for monitoring PA. Further replication, as suggested by Bull et al. [32], has been undertaken and a fair to moderate validity in three languages for use in the European context was found [33]. The Short Form FFQ was validated in 2016 [34] and has been shown as an effective method for assessing diet quality; specifically, for participants based within the UK [34]. Its purpose is to measure diet quality by assessing the intake of 20 food items; including: fruit, vegetables, oily fish, fat, and non-milk extrinsic sugar (NMES). These foods reflect the five dietary components that are recognised as indicators of a healthy diet [35] and are analysed to generate an overall diet quality score (DQS) ranging from 5–15. A low DQS (ranging from 5–10) represents a poor dietary intake, an optimum healthy dietary intake, and DQS was 11. A range between 12–15 represents an adequate dietary intake [31]. The DQS is calculated via the “nutritools” excel spreadsheet [31]. This excel spreadsheet was called the Diet and Nutrition Tool for Evaluation (DANTE) (see Table 1 for the recommended cuts offs for each food group in terms of the number of servings per day/week from each food group [34]). By using DANTE [34], each participant was allocated a score for each of the food groups (see Table 1.). The allocated food group score was out of 3 for each food group. The lowest score allocation per food group could be 1 and the highest could be 3. The recommended score allocation varied according to the food group. For fruit, vegetables, and oily fish, the recommended score allocation was a high score of 3. For the fat and NMES food groups, the recommended score allocation was a low score of 1 (the recommended score allocation is highlighted in italics in Table 1). The DQS, based on fruit, vegetable, oily fish, NMES, and fat intake has been previously recommended as a useful tool for ranking diet quality [34].

The lead researchers designed the questionnaire by merging the GPAQ [30] and the Short Form FFQ [31]. The questions relating to alcohol were deemed inappropriate and, subsequently, removed from the Short Form FFQ due to the age of the older adolescents being under the (UK) legal age to drink. Both questionnaires included rating scale style questions, as well as closed questions that were used to understand the older adolescents’ current food intake and PA habits. To obtain more in-depth answers from the participants, open-ended style questions were also included and focused on self-reported daily PA, engagement in active transport, and active leisure time. Additional open-ended questions focused on how the older adolescents felt healthy lifestyles were promoted to them and to obtain their views and opinions on how these could be promoted to their age group in the future. All questions were piloted with a group of 16–18 years olds that were from similar backgrounds and not participating in the study. No issues or concerns were raised, and the questionnaire was therefore deemed appropriate for use.

The questionnaire was prepared for online use and distribution using Google Documents and Google Forms. All 3 headteachers agreed to allow the study to be undertaken virtually within their schools and the participant consent form and information sheet were at the start of the questionnaire. All participants needed to sign the consent form, agreeing to their answers being included in the research, prior to commencing the survey. They were also required to agree to their data being used for both the research assistants write up reports for their Nuffield Foundation Research Placement, as well as for wider dissemination in research publication/s.

All data collected was pseudonymised and stored via password-protected mechanisms, only accessible to the lead researcher, and in line with GDPR protocol. The research assistants accessed a simplified, anonymous summary version of the data collected via Google Forms. All participants were given the option to withdraw from the study at any time without giving a reason and their results were removed. Personal demographic details of sex were asked and recorded to support analysis. Ethical approval was granted from Canterbury Christ Church University Research Ethics Committee (ETH2021-0243), as well as being approved by Nuffield Foundation Research Placement Programmes. To prevent selection bias [36], all responses that were received were included within the data analysis if all answers within the questionnaire were completed. A short time frame was set for completion of the questionnaire (3 weeks) in an attempt to focus older adolescents on their current knowledge and experiences.

### 2.4. Data Analysis

**Figure 1:**
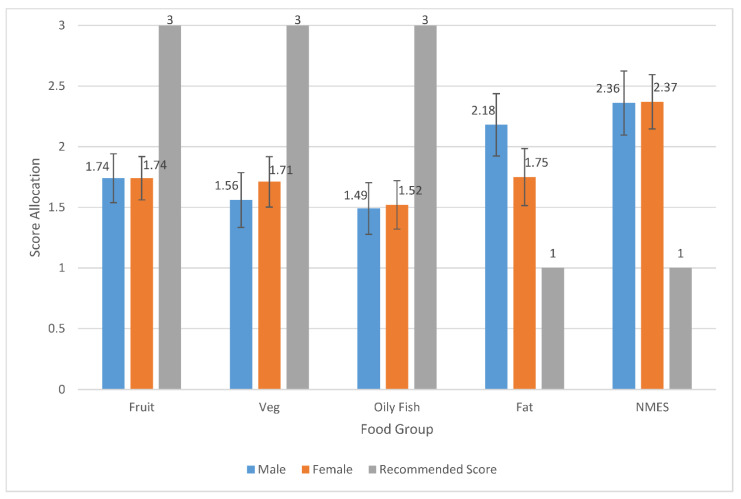
Mean food groups score allocation from the Short Form FFQ, evaluated using DANTE tool per sex compared to the recommended score for each of the food groups.

**Figure 2:**
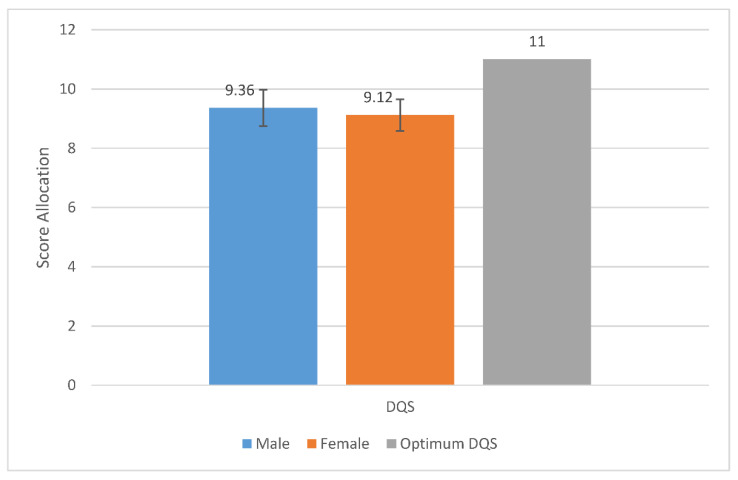
Mean DQS from the Short Form FFQ, evaluated using DANTE tool per sex compared to the optimum DQS.

**Figure 3:**
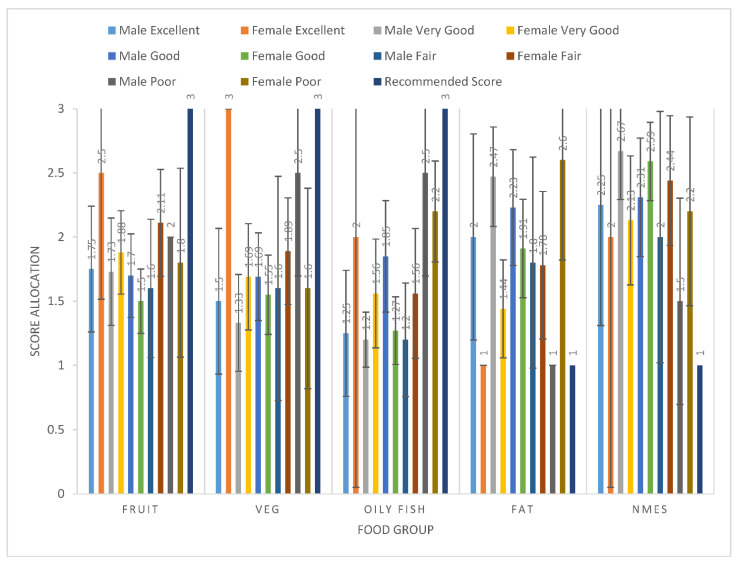
Mean food groups score allocation from the Short Form FFQ, evaluated using DANTE tool per sex and perceived healthiness levels compared to the recommended food groups scores.

The participants reported the frequency that they ate at least one portion of the 20 food items within the Short form FFQ per day/per week. The portion size was explained, e.g., a handful of grapes, an orange, a handful of carrots, a palm-sized amount of side salad, a slice of bread, and a glass of fluid. They chose the following portion per day/per week options: rarely/never, less than once a week, once a week, 2–3 times a week, 4–6 times a week, 7+ times a week; 1–2 times a day, 3–4 times a day, 5+ a day. These were then coded within the DANTE, and if participants did not respond to a food item they were coded as ‘no response’ (as advised by the DANTE tool). The Short Form FFQ questionnaire responses for the frequencies of each 20 food items were then grouped within the DANTE excel spreadsheet according to the 5 food groups (fruit, vegetable, oily fish, fat, NMES) and a score was allocated for each of the food groups (possible scores for each food group could be between 1 and 3), as well as a total DQS calculated (possible DQS could be between 5 and 15) (see Table 1, Figure 1, Figure 2 and Figure 3).

As previously stated, the recommended food group score allocation for fruit, vegetables, and oily fish was 3 and the recommended score for fat and NMES was 1 (highlighted in italics in Table 1). The food group scores were then added together to produce a total DQS. The participants’ food group scores and DQS were then further analysed according to demographics: sex and perceived healthiness (see Figure 3). Perceived healthiness was predicted by participants rating their overall health on a 5-point scale (excellent, very good, good, fair, or poor) over the past 12 months. A multi-variance of statistical analysis (MANOVA) was then undertaken within SPSS 24.0 statistical analysis (IBM Corp, Armok, NY, USA) to assess the group differences across multiple dependent variables of the food group scores and overall DQS. Statistical significance was set at <0.05. The qualitative open-ended questions were analysed via thematic analysis [37].

The GPAQ had analysis guidance provided [38]. These were followed as part of the data analysis process, specifically the cleaning of the data. The questions were then further analysed individually according to demographics: sex, and perceived healthiness. Univariate analysis of variance (ANOVA) was then undertaken for each question to assess the group differences per element of PA previously identified [21] (habitual PA, sedentary time, active transportation, and active leisure). Statistical significance was set at <0.05.

## 3. Results

No significant differences were found by sex for fruit, (F = 2.836, *p* = 0.097); for vegetables; (F = 2.519, *p* = 0.118); for oily fish, (F = 0.29, *p* = 0.866); for fat, (F = 0.168, *p* = 0.683), for NMES, (F = 0.202, *p* = 0.655). Neither males nor females scored the recommended score for any of the food groups, representing poor dietary intake for all food groups [31] (see Figure 1).

No significant differences were found for sex (F = 1.328, *p* = 0.254). Neither males nor females scored the optimum DQS. Both sexes scored a low DQS (with a low specified as ranging between 5 and 10) representing an overall poor dietary intake [31] (see Figure 2).

Figure 3 illustrates the participants’ mean food group score allocation from the Short Form FFQ, evaluated using the DANTE tool, according to their demographics of sex and perceived healthiness. Perceived healthiness was predicted by participants’ rating their overall health on a 5-point scale (excellent, very good, good, fair, or poor) over the past 12 months.

**Figure 4:**
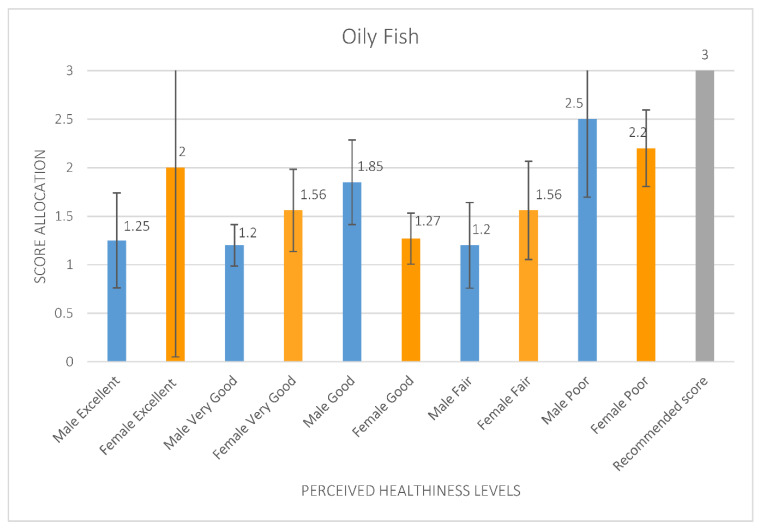
Mean oily fish score allocation from the Short Form FFQ evaluated using DANTE tool per perceived healthiness levels for all older adolescents compared to the recommended oily fish food group score.

There were significant main effects found for perceived levels of healthiness and intake of oily fish (F = 2.317, *p* = 0.066), with those who regarded themselves in the last 12 months as having poor levels of healthiness found to be eating the most and closest to the recommended score (2.29, 95% CI [2.19, 2.29]) for oily fish. Yet, those who rated themselves as having very good levels of healthiness ate considerably lower levels of oily fish, scoring only 1.39 (95% CI [1.25, 1.53]) on the DANTE tool (see Figure 4).

**Figure 5:**
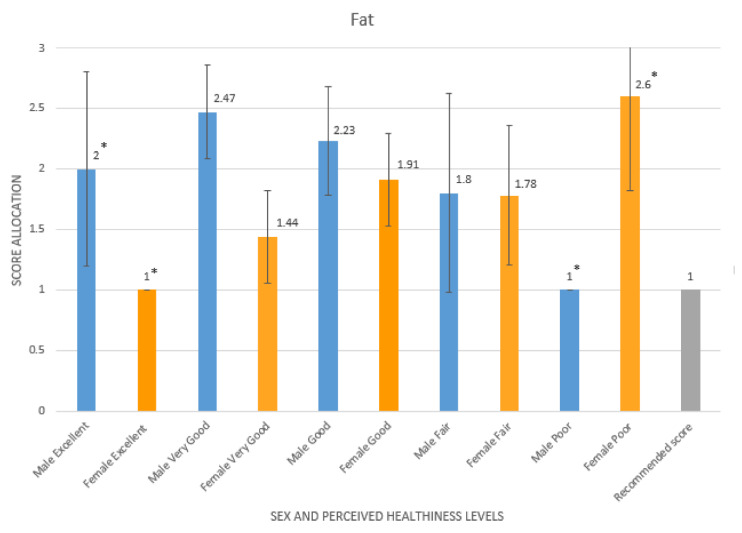
Mean fat food group score allocation from the Short Form FFQ evaluated using DANTE tool per sex and perceived healthiness levels compared to the recommended fat food group score (* indicates significant differences).

There were significant interactions between sex, perceived healthiness levels in the past 12 months, and fat scores (F = 2.532, *p* = 0.048). The data indicates that among males, those who rated themselves as having poor health had eaten the recommended intakes of fat (1.00, 95% CI [1, 1]).This is in comparison to females who rated themselves as having poor health but ate more than the recommended intake of fat (2.60, 95% CI [2.42, 2.78]). The data also indicates that among males, those who rated themselves as having excellent health ate more than the recommended intake of fat (2.00, 95% CI [1.2, 2.8]). This is in comparison to females who rated themselves as having excellent health and who ate the recommended intake of fat (1.00, 95% CI [1, 1]) (see Figure 5). This data (F = 8.151, *p* = 0.029) indicates a mismatch between perceived healthiness and eating the recommended dietary intake for fat, particularly for males.

**Figure 6:**
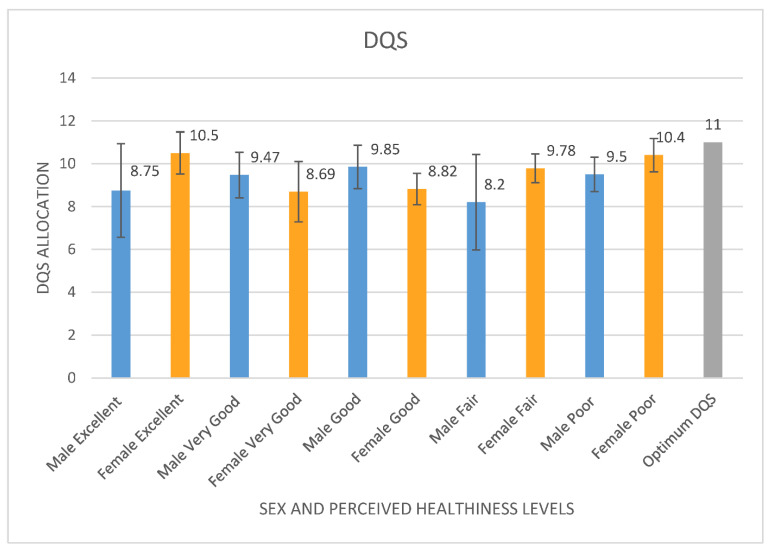
Mean DQS from the Short Form FFQ evaluated using DANTE tool per sex and perceived healthiness levels compared to the optimum DQS.

No significant differences were found between sex and perceived healthiness (F = 3.747, *p* = 0.935). Both sexes and all perceived healthiness levels scored a low DQS (with a low specified as ranging between 5 and below 11), representing an overall poor dietary intake [31]. Females with an excellent perceived level of healthiness (10.5) and females with a poor perceived level of healthiness (10.4) were the closest to scoring the optimum DQS of 11. The data indicate that levels of perceived healthiness and sex did not have an impact on reaching the optimum DQS (see Figure 6).

### 3.1. Physical Activity and Sedentary Time

The PA data analysis focused on self-reported daily PA, including intensity levels in work/study settings, as well as the number of days these intensity levels occurred per week. This analysis also focused on their engagement with active transport within a typical week, as well as their reported frequency and intensity of participation in active leisure time, such as sport and fitness. For sedentary time, the number of minutes engaged in sedentary activities such as sitting, or reclining was recorded. Examples from the questionnaire included: sitting whilst moving to and from places (such as traveling in a car, bus, or train); sitting at a desk; sitting with friends; sitting whilst reading; playing cards or screen time, (such as watching television). There were no significant findings for any of the PA data with sex or perceived healthiness.

Overall, 60% (*n* = 56) reported that they undertook and reached the recommended 60 min or more of MVPA per day, whilst 40% (*n* = 37) did not meet these recommended daily PA targets [39]. When work or study settings were explored, 59% (*n* = 55) reported that their work or study settings included moderate intensity levels, described in the questionnaire as those that caused small increases in breathing or heart rate, whilst, 28% of adolescents (*n* = 26) reported that their work or study settings involved vigorous intensity levels that caused large increases in breathing or heart rate. The participants reported high levels of active transport in that 92% (*n* = 86) stated that they walked or used a bicycle for at least 10 minutes continuously to go to and from places, with 86% (*n* = 80) reporting this use to be on 5 days or more per week.

There was almost an even split in those reporting they undertook vigorous-intensity active leisure as sport, fitness, or recreational leisure activities that caused large increases in breathing or heart rate, such as running or football, for at least 10 minutes continuously (51%; *n* = 47). A total of 49% (*n* = 46) reported that they did not undertake this type of activity.

Close to half of all participants (47% (n = 44) reported being sedentary for between 5 and 8 h per day, with only 26% (*n* = 24) reporting being sedentary for under 4 h per day. Nineteen percent (*n* = 18) reported being sedentary for more than 8 h a day and eight percent (*n* = 7) of adolescents reported not knowing how long they were sedentary per day.

### 3.2. Promotion of Healthy Lifestyles

The viewpoints of older adolescents towards how healthy lifestyles are promoted to them are presented via quotations; a recommended technique [26], which offers a representation of current experiences [40]. The viewpoints have been coded via thematic analysis [37] into negative and positive experiences (see Table 2 and Table 3). There were, overall, more negative compared to positive viewpoints expressed.

The most popular response, when asked about whether healthy lifestyles were promoted to them, was that they “did not feel that healthy lifestyles were promoted to them directly”. They also expressed that they felt healthy lifestyles were portrayed inaccurately, with several participants also stating that they did not know how healthy lifestyles were promoted at all. Their responses are illustrated in Table 2 and also highlight the financial impact of being healthy, as well as the impact of social media and fake news.

There were only some positive responses that indicated the participants felt that healthy lifestyles were promoted well to them. These were from a range of voices, including doctors, social media, the government, and schools, particularly within Physical Education lessons (Table 3).

## 4. Discussion

This study aimed to examine older adolescents’ knowledge and understanding of PA recommendations and nutrition guidelines and their current thoughts relating to the promotion of these lifestyle behaviours to their age group. Although adolescence is a life stage that can harbour risks and dangers, it also presents a great opportunity for health and wellbeing to be promoted sustainably via appropriate education and preventive initiatives [16]. Yet, findings from this research reveal that older adolescents experience barriers to accessing health information; specifically those relating to PA and healthy eating. This supports previous work [14] and aligns with the suggestion that adolescence is the forgotten age phase of targeted health promotion efforts [2]. Today’s young people are the future adults and leaders of society, and these findings support the argument that the promotion of health during adolescence should be considered one of the most important opportunities to educate and prevent future ill-health [16]; only then will the full potential of future generations be met.

### 4.1. Food Frequencies

Previous research has identified that adolescent diets are poorly understood [41]. The current study found similar results and revealed that older adolescents did not eat the recommended amount for each of the food groups, nor achieved the recommended DQS (Figure 1 and Figure 2). The participants also revealed a relatively negative experience of healthy lifestyle promotion targeted at them (Table 2).

Appropriate nutrition can have a profound impact on the current and future health of adolescents (ages 10–19 years) [42]. Yet, findings from this study support data gathered worldwide that continue to report adolescents infrequently consume fruit and vegetables [41]. Further, the data indicate that many participants were not eating the recommended servings/amounts of other food groups (fat, oily fish) (Figure 4 and Figure 5). Given that these participants also showed a disconnect between perceived level of healthiness and eating the recommended amounts (Figure 3) suggests that these adolescents poorly understand current food intake recommendations. This was evident in the finding that among those who reported poor levels of healthiness, their oily fish intakes were the closest to what is recommended; whilst those who rated themselves as having very good levels of healthiness reported consuming considerably lower levels than the recommended amounts for oily fish. When analysed by sex, this disconnect was only found for males (Figure 4). Those who rated themselves as having poor healthiness reported eating the recommended amounts of fat, compared to those females who also reported having a poor rating for healthiness but ate in excess of the recommended amounts for fat (Figure 5). A misconception as to “what is healthy” is therefore emerging from the data for males. Future research should look to gain a deeper insight into the health literacy of adolescent males, as previous research found lower baseline health literacy to be associated with a lower self-rating of general health, and an unhealthier diet among young people in this age group [43].

No participant’s dietary intake met the recommended DQS [37] (Figure 6). This may be due to a multitude of factors [44], with lack of understanding or cost barriers associated with healthy food consumption being two examples. Affordability is reported as one of the leading barriers to improving nutritional quality in populations [45], which aligns with the findings in this current study which found some participants reporting that they were prevented from undertaking healthy lifestyles due to the financial burden associated with it. These responses may be linked to the older adolescents’ economic background, as all the participating schools were based within low socio-economic parts of the country. Yet, all three of the schools did comply with the Government’s healthy eating guidelines for their canteen food [29] and have a curriculum focus on “how to maintain healthy eating” [4] (p. 37), so it remains unclear why these older adolescents’ food intakes are not somewhat closer to meeting the recommended intakes for health. Further in-depth qualitative data are needed to ascertain why this is the case and to ascertain why the school environment and associated policies are not leading to better dietary choices among this age group.

### 4.2. Physical Activity

The UK Chief Medical PA Guidelines [46] state that children and young people (5–18 years) should be aiming for an average of at least 60 min of MVPA per day, across the week. Globally, however, most adolescents do not meet PA guidelines [47] and this current study supports these findings, with only 60% (three in five) reporting reaching the recommended levels. Unfortunately, the adolescent life stage is poorly understood in PA research [48]. Further insight is needed, particularly as it was recently outlined these young people are one of the least likely populations to have the support needed to meet PA recommendations for health [49]. In response, researchers [48] have called for urgent policy development to support PA behaviors in adolescents.

There were no significant differences found for PA by sex in the current study, indicating that no one target demographic needs additional support; it is a universal need. This conflicts with previous research that has consistently found boys to be more active than girls [47,50]. Interestingly, the adolescents in the current study also reported digital media as having both negative and positive impacts on their PA levels (and dietary intakes), despite research stating that the digital environment provides a potential solution to enhancing these behaviours in the adolescent population [51]. Clearly, important knowledge gaps continue to exist. Efforts are needed to better understand adolescent PA in order to improve intervention implementation and policy development [49]. Researchers [47] suggest more engagement and dialogue are needed with adolescents; only then can we ensure that all older adolescents are supported in reaching their daily PA targets more effectively.

Reducing sedentary behaviour in all age groups is one of the focus areas of the World Health Organization’s [42] mission statement. Sedentary behaviour is one of the primary risk factors for non-communicable diseases, including heart disease, stroke, cancer, and diabetes [52]. If, however, we can increase PA, evidence suggests we can reduce “disease burden and overall mortality, as well as promoting wellbeing and mental health for all” [42] (p. 52). Some of the adolescents noted in the comments box of the questionnaire that sedentary behaviour occurred a great deal during their school time. This is despite continued suggestions [42] that education facilities should be built so they “reduce sedentary behaviour to create better learning environments for all” [42] (p. 53). Overall, 66% (*n* = 61) of the older adolescents reported being sedentary for 5 h or more and a further 7% (*n* = 7) stated they did not know their levels of this behaviour. Clearly, changes to the school curriculum are needed but more opportunities for PA before, during, and after school, must also be explored [53].

Worldwide levels of inactivity can be as high as 70% due to changing patterns of transportation [42]. Active transportation to/from school offers a promising opportunity to reduce these levels by integrating PA into adolescents’ routines [54]. In a comparison of 49 countries, the global average of children and youth engaging in active transportation was a “C”, which represents approximately 47–53%, or about half, of global youth engaging in non-motorised travel modes such as walking and/or cycling [55]. In England, data suggests that 43% (C-) of adolescents engage in active transport regularly [56]. Results from this current paper, however, contradict these findings, with 92% reporting they undertook walking or cycling for at least 10 min continuously per day, and of these, 86% report this to occur at least 5 times a week. Although the reasons for this conflicting finding are unknown, one suggestion put forward is the variability in the definition of active transportation globally [55]. The high percentages reported in the current study may be also due to the participants having limited access to individual driving licences, or driving tests, during the data collection period. The COVID-19 pandemic meant due to social restrictions and lockdowns, driving licences other than with family members were not permitted and driving tests did not take place. In January 2022, it was reported that there was still a 10-month delay in learner drivers being able to take their practical exams; exacerbated by the shortage of examiners and instructors [57]. As a result, these older adolescents may have had limited access to motorised independence during this time and walking and/or cycling may have been their only means of independent transport. Further research is needed to see if this level of active transport continues once the pandemic eases and to unpack the understanding behind active transportation to ascertain whether these older adolescents see it as part of their daily PA, or “just” the way they maintain their independence and move to places such as school/study and work settings. Further, more comprehensive surveillance that incorporates duration, distance, frequency, direction, and other destinations than school could improve this understanding [55].

### 4.3. Promoting Healthy Lifestyles

Investing in adolescent health and well-being creates dividends in the drive to create healthier, more sustainable societies [50]. In the World Health Organization’s global accelerated action for the health of adolescents (AA-HA!) guidance [58], a systematic approach to planning and implementing national programs that address the specific needs of adolescents has been called for. This is in response to research [59] that found brief education interventions to increase knowledge of, for example, the sugar content of food and drink, but which did not extrapolate into behaviour change or decreases in intake of these foods in Scottish children [59]. Adolescents are less likely to be motivated to change their current behaviour in order to reap health benefits in the future [41], so these interventions must be longitudinal and creative; harnessing widely shared adolescent values in order to successfully promote healthy lifestyles in this population.

Similar suggestions have been made by researchers [60] evaluating the ‘Food Dudes’ programme, which was run in 15 primary schools in the West Midlands, England. The authors called for the whole school environment to be invested in the programme, as they found the educational programme to have a limited effect in decreasing the consumption of high fat and high sugar foods at lunchtime among participants. These findings are disconcerting, given that the majority of healthy lifestyle promotion interventions to date have been aimed at the primary school age group. If interventions are not working at this young age, by the time these children move to adolescence, when habitual behaviours may have already been formed [61], the window for opportunity to intervene and change behaviour may be more difficult. By older adolescence, educational establishments may also even assume that these young people already have the knowledge to make informed choices about their PA and nutrition. This is illustrated in this study by the response, “It’s presumed we all know how to eat better and be healthy” and 80 participants (90%) did not report their schools as places that supported them in their healthy lifestyles or dietary choices. Assumptions cannot, therefore, be made about the knowledge and understanding of healthy lifestyles for this age phase [62] and further research is needed to ascertain where any particular misconceptions around healthy lifestyles may lie.

As social media use is widespread in adolescence, its potential as a health communication tool shows promise [63,64]. The participants of the current study reported social media as one method by which healthy lifestyles were promoted to them. It has been previously suggested the development of health literacy, through social media, is a potential way to support adolescents’ understanding of healthy lifestyles [65]. Yet, this information must also be reliable and incorporate attractive messages tailored to meet the diverse needs of adolescents [64]. Within the current study, seven participants (8%) felt that healthy lifestyles were promoted as either “ok” or “well” to them. However, 38 participants (41%) did not feel it was promoted or directed to them and some (*n* = 5, 5%) even warned they felt this type of promotion was potentially harmful as it exacerbates body issues. Therefore, although social media has huge potential to increase health knowledge and awareness among adolescents [66], more needs to be known about the effectiveness of social media platforms at promoting health [64,67] before it can be encouraged for widespread use as a health behaviour change tool for this age group.

## 5. Strengths and Limitations

This study is timely due to its ability to examine the impact of the new English curriculum and policy [4] implemented within secondary (high school) settings, which is aimed at making older adolescents (16–18-year-olds) ready for healthy adulthood [20]. This study is the first of its kind to be able to comment, from an adolescents’ perspective, on how the curriculum within these three schools has influenced their knowledge and understanding of healthy lifestyles. The findings could inform a larger study examining more schools from a wider range of different economic and locational settings. Although further research is still needed, another strength of the current study is its novel insight, from the adolescents’ point of view, into how effective social media is at promoting health to this age group. To date, too few studies have investigated these platforms [67].

The sample size of participants (*n* = 93) in this study was also a strength, as each school’s response rate was higher than the average class size for secondary schools (21.7), according to National Statistics in England [27]. When compared to previous health promotion UK-based research [68,69] the sample size is also larger. Yet, it is important to note this comparison to previous health promotion research within the UK specifically relates to the sample size, rather than directly comparing the results. The previous studies were all undertaken among young adults and adults aged between 18–30 years. There is, to date, no previous PA and nutrition knowledge and understanding research within the UK that has focused solely on older adolescents. In Scotland, ref. [68] the previous research focused on health promotion with a sample size of only 19, whilst research in the northeast of England [69] had a sample size of 54, which was based on young adults (18–25-year-olds) in the northeast of England. Only one study that focused on the evaluation of healthy eating leaflets modified from the British Nutrition Foundation used a slightly higher sample number of 104 [70], but this again was based on adults ≥ 18 years of age. It is acknowledged though that the sample in the preliminary study selected was not fully random or stratified [71] and therefore only reflects the population of the three schools, rather than the population as a whole [72].

Data collection took place during the COVID-19 pandemic, which may have been considered a difficult time and a potential limitation to this study, as we were discussing healthy lifestyles when restricted movements and limited social interaction were in place. However, the Department of Education [73] reported on the state of the nation, in which they consider children and young people’s well-being and found that despite the ongoing pandemic, older adolescents reported their health on average as “very good” (p. 55), despite increases in restlessness and attention difficulties (p. 118). Therefore, it could be argued that our data are a timely strength of the study and highlights the importance of asking such questions during unique times of a global pandemic.

The use of only three schools within the sample is recognised as a limitation, due to the generalisability of the research. Yet, the project was set up with links to the Nuffield Foundation Research Placement and therefore had a purposeful focus on the sample schools, with a research assistant involved from each of the schools in order to make the research meaningful to those undertaking the research placement. The research project was also designed as a preliminary study to examine if there was a potential need for more targeted PA and nutrition education for older adolescents. For future research, as stated above, upscaling and increasing the larger sample of schools and adolescents would be recommended. In addition, a wider sample size could be used that are from broader socioeconomic backgrounds. This would also facilitate collecting data on the implication of cost on healthy lifestyles that were found in the qualitative responses in the current study. It is also important to acknowledge that some found the PA questions to be difficult to answer, which previous researchers [74] have noted can be a highly complex cognitive task that some may struggle with. Further piloting of questions included in the questionnaire is therefore needed before any future iterations of the study commence.

## 6. Conclusions

This research has illustrated that English older adolescents do not feel that they are targeted in terms of current healthy lifestyle promotion. They also report not being taught this at school, particularly in relation to PA and nutrition, despite explicitly being required to receive education focusing on healthy lifestyles as per their curriculum [4]. To instil healthy lifestyle behaviours during this core key life stage, targeted PA and nutrition education is warranted that also includes the adolescent voice. Further, gaining a deeper insight into male older adolescents’ health literacy is needed to ensure they fully understand, appraise and use information and services to make decisions about their health now that will benefit them as they transition into adulthood. This is in addition to the need for more specific policy calls for this specific age phase, to prevent our older adolescents from being forgotten in the future.

## Figures and Tables

**Table 1:** DANTE score allocation per food group (Cleghorn and Cade, 2017).

Food Group	Score Allocated		
	1	2	3
**Fruit**	<=2 servings/wk	>2 servings/wk and <2 servings/day	*>=2 servings/day*
**Vegetables**	<=1 servings/day	1–3 servings/day	*>=3 servings/day*
**Oily Fish**	No intake	0–200 g/wk	*>=200 g/wk*
**Fat**	*>=1 ½ × UK recommendations (127.5 g/day)*	1–1 ½ × UK recommendations	<=UK recommendations (85 g/day)
**NMES**	*>=1 ½ × UK recommendations (90 g/day)*	1–1 ½ × UK recommendations	<=UK recommendations (60 g/day)

**Table 2:** Negative responses to healthy lifestyle promotion.

“They aren’t promoted enough for our age group and no time in school is given to educate us about health or nutrition.”
“I’m really sceptical about the healthy lifestyles that I’ve seen, they’re either selling some fake product that’ll burn your fat or try to make you do things that aren’t necessarily right.”
“I feel that healthy lifestyles are marketed towards young people my age in a very disillusioning way, as it makes us think we need to spend money to get fit.”
‘I feel I have been left in the dark and left alone’
Healthy lifestyles were promoted to them “as children….but as we’ve gotten older, it’s all stopped”.
“It’s presumed we all know how to eat better and be healthy”
“Healthy lifestyles are not promoted enough, so I don’t know a lot about my own health and fitness”,
“There are no healthy options to eat at school, not even an apple!”
“It’s not promoted, and it is hard to do any fitness on top of school or work as I have a 40 min walk to and from school and this leaves me drained”
“Not very well”
“Not promoted at all for our age group”
“I want to be able to eat chips and not feel fat”
“Unhealthy lifestyles are promoted in the form of size 0 models in magazines”
“Only promoted in ways that exacerbates body issues
“There is a lack of promotional material about healthy lifestyles of us, we need cheaper gym access.”

**Table 3:** Positive responses to healthy lifestyle promotion.

“Government recommended advice is always shared and schools gives us talks on how we can be healthier”
“Commonly promoted on social media”
“Promoted well”
“Spoken about within Physical Education lessons within school and emphasised repeatedly”
“By doctors”
“By drilling us with the importance of eating healthy and doing physical activity”
“By banning sugary drinks and snacks at school has tried to instil that it’s not good to be eating junk food every day at school”
“Via technology e.g. my apple watch”

## Data Availability

The data presented in this study are available on request from the corresponding author.

## References

[1] Viner R., Macfarlane A. (2005). Health promotion. Br. Med. J..

[2] Tylee A., Haller D.M., Graham T., Churchill R., Sanci L.A. (2007). Youth-friendly primary-care services: How are we doing and what more needs to be done?. Lancet.

[3] World Health Organization Europe (2020). “Nothing About Us, without Us” Tips for Policy Makers on Child and Adolescent Participation in Policy Development.

[4] Department for Education (DfE) (2019). Relationships Education, Relationships and Sex Education (RSE) and Health Education. Statutory Guidance for Governing Bodies, Priorietors, Head Teachers, Principals, Senior Leadership Teams, Teachers.

[5] Higgins J.W., Begoray D., MacDonald M. (2009). A Social Ecological Conceptual Framework for Understanding Adolescent Health Literacy in the Health Education Classroom. Am. J. Community Psychol..

[6] Smith K., Wells R., Hawkes C. (2022). How Primary School Curriculums in 11 Countries around the World Deliver Food Education and Address Food Literacy: A Policy Analysis. Int. J. Environ. Res. Public Health.

[7] Department for Education (DfE) (2013). Design and Technology Programmes of Study: Key Stages 1 and 2.

[8] Berkman N.D., Davis T.C., McCormack L. (2010). Health Literacy: What Is It?. J. Health Commun..

[9] Nutbeam D. (2009). Defining and measuring health literacy: What can we learn from literacy studies?. Int. J. Public Health.

[10] van der Heide I., Wang J., Droomers M., Spreeuwenberg P., Rademakers J., Uiters E. (2013). The relationship between health, education, and health literacy: Results from the Dutch Adult Literacy and Life Skills Survey. J. Health Commun..

[11] National Health Service (NHS) (2015). Enabling People to Make Informed Health Decisions. https://www.england.nhs.uk/ourwork/patient-participation/health-decisions/.

[12] Schulenkorf T., Sørensen K., Okan O. (2022). International Understandings of Health Literacy in Childhood and Adolescence—A Qualitative Explorative Analysis of Global Expert Interviews. Int. J. Environ. Res. Public Health.

[13] Berkman N.D., Sheridan S.L., Donahue K.E., Halpern D.J., Crotty K. (2011). Low health literacy and health outcomes: An updated systematic review. Ann. Intern. Med..

[14] Nutbeam D. (2008). The evolving concept of health literacy. Soc. Sci. Med..

[15] Bröder J., Okan O., Bauer U., Bruland D., Schlupp S., Bollweg T.M., Saboga-Nunes L., Bond E., Sørensen K., Bitzer E.M. (2017). Health literacy in childhood and youth: A systematic review of definitions and models. BMC Public Health.

[16] Kleinert S. (2007). Adolescent health: An opportunity not to be missed. Lancet.

[17] Lim S.S., Vos T., Flaxman A.D., Danaei G., Shibuya K., Adair-Rohani H., AlMazroa M.A., Amann M., Anderson H.R., Andrews K.G. (2012). A comparative risk assessment of burden of disease and injury attributable to 67 risk factors and risk factor clusters in 21 regions, 1990–2010: A systematic analysis for the Global Burden of Disease Study 2010. Lancet.

[18] Wang X., Ouyang Y., Liu J., Zhu M., Zhao G., Bao W., Hu F.B. (2014). Fruit and vegetable consumption and mortality from all causes, cardiovascular disease, and cancer: Systematic review and dose-response meta-analysis of prospective cohort studies. BMJ.

[19] Public Health England (2016). National Diet and Nutrition Survey: Results from Years 5 and 6 (Combined) of the Rolling Programme (2012/2013–2013/2014).

[20] World Health Organization Europe (2021). Adolescents Taking the Lead. Multistakeholder Consultation to Promote Adolescent Well-Being in the WHO European Region.

[21] Neumark-Sztainer D., Story M., Toporov E., Himes J.H., Resnick M.D., Blum R.W. (1997). Covariations of eating behaviors with other health-related behaviors among adolescents. J. Adolesc. Health.

[22] Milligan R.A., Burke V., Dunbar D.L., Spencer M., Balde E., Beilin L.J., Gracey M.P. (1997). Associations between lifestyle and cardiovascular risk factors in 18-year-old Australians. J. Adolesc. Health.

[23] Senderowitz J. (1995). Adolescent Health: Reassessing the Passage to Adulthood.

[24] Jessiman P.E., Campbell R., Jago RVan Sluijs E.M.F., Newbury-Birch D. (2019). A qualitative study of health promotion in academy schools in England. BMC Public Health.

[25] Office for National Statistics (2011). Census for England and Wales.

[26] Patton M.Q. (2002). Qualitative Research and Evaluation Methods.

[27] Department for Education (DfE) National Statistics (2019). Schools, Pupils and Their Characteristics: January 2019.

[28] Healthy Schools Initiative (2009). Physical Activity Guidance Documents.

[29] Public Health England (2016). Government Dietary Recommendations. Government Recommendations for Energy and Nutrients for Males and Females 1—18 Years and 19+ Year.

[30] World Health Organization (2004). Global Physical Activity Questionnaire (GPAQ).

[31] Cleghorn C., Cade J. (2017). Short Form Food Frequency Questionnaire. https://www.nutritools.org/tools/136#t1.

[32] Bull F.C., Maslin T.S., Armstrong T. (2009). Global physical activity questionnaire (GPAQ) nine country reliability and validity study. J. Phys. Act. Health..

[33] Wanner M., Hartmann C., Pestoni G., Winfried Martin B., Siegrist M., Martin-Diener E. (2017). Validation of the Global Physical Activity Questionnaire for self-administration in a European context. BMJ Open Sport Exerc. Med..

[34] Cleghorn C., Harrison R.A., Ransley J.K., Wilkinson S., Thomas J., Cade J.E. (2016). Can a dietary quality score derived from a short-form FFQ assess dietary quality in UK adult population survey?. Public Health Nutr..

[35] World Health Organization (2003). Diet, Nutrition and the Prevention of Chronic Diseases.

[36] Berg K.E., Latin R.W. (2008). Essentials of Research Methods in Health, PE, Exercise Science and Recreation.

[37] Braun V., Clarke V., Cooper H., Camic P.M., Long D.L., Panter A.T., Rindskopf D., Sher K.J. (2012). Thematic analysis. APA Handbook of Research Methods in Psychology, Vol. 2. Research Designs: Quantitative, Qualitative, Neuropsychological, and Biological.

[38] World Health Organization (2004). Global Physical Activity Questionnaire (GPAQ) Analysis Guide.

[39] World Health Organization (2010). Global Recommendations on Physical Activity for Health.

[40] Eldh A.C., Årestedt L., Berterö C. (2020). Quotations in Qualitative Studies: Reflections on Constituents, Customs and Purpose. Int. J. Qual. Methods.

[41] Beal T., Morris S.S., Tumilowicz A. (2019). Global Patterns of Adolescent Fruit, Vegetable, Carbonated Soft Drink, and Fast-Food Consumption: A Meta-Analysis of Global School-Based Student Health Surveys. Food Nutr. Bull..

[42] World Health Organization (2018). Guideline: Implementing Effective Actions for Improving Adolescent Nutrition.

[43] Park A., Eckert T.L., Zaso M.J., Scott-Sheldon L.A.J., Vanable P.A., Carey K.B., Ewart C.K., Carey M.P. (2017). Associations Between Health Literacy and Health Behaviors Among Urban High School Students. J. Sch. Health.

[44] Corfe S. (2018). What Are the Barriers to Eating Healthily in the UK?.

[45] Giacobone G., Tiscornia M.V., Guarnieri L., Castronuovo L., Mackay S., Allemandi L. (2021). Measuring cost and affordability of current vs. healthy diets in Argentina: An application of linear programming and the INFORMAS protocol. BMC Public Health.

[46] UK Chief Medical Officers (2019). Physical Activity Guidelines. https://assets.publishing.service.gov.uk/government/uploads/system/uploads/attachment_data/file/1054282/physical-activity-for-children-and-young-people-5-to-18-years.pdf.

[47] Guthold R., Stevens G.A., Riley L.M., Bull F.C. (2020). Global trends in insufficient physical activity among adolescents: A pooled analysis of 298 population-based surveys with 1·6 million participants. Lancet Child Adolesc. Health.

[48] van Sluijs E.M., Ekelund U., Crochemore-Silva I., Guthold R., Ha A., Lubans D., Oyeyemi ALDing D., Katzmarzyk P.T. (2021). Physical activity behaviours in adolescence: Current evidence and opportunities for intervention. Lancet.

[49] (2021). The Lancet (2021) Physical Activity 2021 Executive Summary. https://www.thelancet.com/series/physical-activity-2021.

[50] World Health Organization Europe (2022). 85% of Adolescent Gilrs Don’t Do Enough Physical Activity: A New WHO Study Calls for Action. https://www.euro.who.int/en/health-topics/disease-prevention/physical-activity/news/news/2022/3/85-of-adolescent-girls-dont-do-enough-physical-activity-new-who-study-calls-for-action.

[51] Rose T., Barker M., Jacob C., Morrison L., Lawrence W., Strömmer S., Vogel C., Woods-Townsend K., Farrel D., Inskip H. (2017). A Systematic Review of Digital Interventions for Improving the Diet and Physical Activity Behaviors of Adolescents. J. Adolesc. Health.

[52] Peters R., Ee N., Peters J., Beckett N., Booth A., Rockwood K., Anstey K.J. (2019). Common risk factors for major noncommunicable disease, a systematic overview of reviews and commentary: The implied potential for targeted risk reduction. Ther. Adv. Chronic Dis..

[53] World Health Organization Europe (2022). WHO/Europe Urges Government to Include Young People in Decisions about Their Health. https://www.euro.who.int/en/health-topics/Life-stages/child-and-adolescent-health/news/news/2022/2/whoeurope-urges-governments-to-include-young-people-in-decisions-about-their-health.

[54] LaRouche R., Saunders T.J., Faulkner G., Colley R., Tremblay M. (2014). Associations between active school transport and physical activity, body composition, and cardiovascular fitness: A systematic review of 68 studies. J. Phys. Act. Health.

[55] González S.A., Aubert S., Barnes J.D., Larouche R., Tremblay M.S. (2020). Profiles of Active Transportation among Children and Adolescents in the Global Matrix 3.0 Initiative: A 49-Country Comparison. Int. J. Environ. Res. Public Health.

[56] Standage M., Sherar L., Curran T., Wilkie H.J., Jago R., Davis A., Foster C. (2018). Results From England’s 2018 Report Card on Physical Activity for Children and Youth. J. Phys. Act. Health.

[57] RAC (2022). Driving Test Backlog Leaves Learners Facing 10-Month Wait. https://www.rac.co.uk/drive/news/infrastructure/driving-test-backlog-leaves-learners-facing-10-month-wait/.

[58] World Health Organization (2017). Global Accelerated Action for the Health of Adolescents (AA-HA!) Guidance to Support Country Implementation. http://apps.who.int/iris/bitstream/handle/10665/255415/9789241512343-eng.pdf;jsessionid=7E005BA31D28787F81D706BFEBCD827F?sequence=1.

[59] Griffin T.L., Jackson D.M., McNeill G., Aucott L.S., Macdiarmid J.I. (2015). A Brief Educational Intervention Increases Knowledge of the Sugar Content of Foods and Drinks but Does Not Decrease Intakes in Scottish Children Aged 10-12 Years. J. Nutr. Educ. Behav..

[60] Upton P., Taylor C., Upton D. (2015). The effects of the Food Dudes Programme on children’s intake of unhealthy foods at lunchtime. Perspect Public Health.

[61] Telama R. (2009). Tracking of physical activity from childhood to adulthood: A review. Obes. Facts..

[62] Gracey D., Stanley N., Burke V., Corti B., Beilin L.J. (1996). Nutritional knowledge, beliefs and behaviour in teenage school students. Health Educ. Res..

[63] Vaterlaus J.M., Patten E.V., Roche C., You-ng J.A. (2015). #Gettinghealthy: The perceived influence of social media on young adults health behaviours. Comput. Hum. Behav..

[64] Plaisime M., Robertson-James C., Mejia L., Núñez A., Wolf J., Reels S. (2020). Social Media and Teens: A Needs Assessment Exploring the Potential Role of Social Media in Promoting Health. Soc. Media Soc..

[65] Levin-Zamier D., Lemish D., Gofin R. (2011). Media Health Literacy (MHL): Development and measurement of the concept among adolescents. Health Educ. Res..

[66] Vandelanotte C., Kirwan M., Rebar A., Alley S., Short C., Fallon L., Buzza G., Schoeppe S., Mahar C., Duncan M.J. (2014). Examining the use of evidence-based and social media supported tools in freely accessible physical activity intervention websites. Int. J. Behav. Nutr. Phys. Act..

[67] Guse K., Levine D., Martins S., Lira A., Gaarde J., Westmorland W., Gilliam M. (2012). Interventions using new digital media to improve adolescent sexual health: A systematic review. J. Adolesc. Health.

[68] Berry E., Aucott L., Poobalan A. (2018). Are young adults appreciating the health promotion messages on diet and exercise?. Z. Gesundh..

[69] Giles E.L., Brennan M. (2015). Changing the lifestyles of young adults. J. Soc. Mark..

[70] Baker H.J., Butler L.T., Chambers S.A., Traill W.B., Lobb A.E., Herbert G. (2010). An RCT study to evaluate a targeted, theory driving healthy eating leaflet. Soc. Sci. Med..

[71] Smith M.F. (2018). Research Methods in Sport.

[72] Gorard S. (2001). Quantitative Methods in Educational Research: The Role of Numbers Made Easy.

[73] Department for Education (DfE) (2020). State of the Nation 2020: Children and Young People’s Wellbeing.

[74] Schneider P.L., Crouter S.E., Bassett D.R. (2004). Pedometer measures of free-living physical activity: Comparison of 13 models. Med. Sci. Sports Exerc..

